# Improvements in quality of life associated with biphasic insulin aspart 30 in type 2 diabetes patients in China: results from the A_1_chieve® observational study

**DOI:** 10.1186/s12955-014-0137-9

**Published:** 2014-11-26

**Authors:** Wenying Yang, Xiaoming Zhuang, Yukun Li, Qing Wang, Rongwen Bian, Jianguo Shen, Eva Hammerby, Li Yang

**Affiliations:** China-Japan Friendship Hospital, Beijing, China; Fu Xing Hospital, Capital Medical University, Beijing, China; The Third Hospital of Hebei Medical University, Hebei, China; Department of Endocrinology, China-Japan Union Hospital of Jilin University, Changchun, China; Jiangsu Provincial Official Hospital, Jiangsu, China; The First Affiliated Hospital, Zhejiang University, Zhejiang, China; Novo NordiskA/S, Copenhagen, Denmark; Department of Health Policy and Management, School of Public Health, Peking University, No 38 Xueyuan Rd. Haidian District, Beijing, 100191 China

**Keywords:** Quality of life, Biphasic insulin aspart 30, Type 2 diabetes, Chinese setting, Observational, EQ-5D

## Abstract

**Background:**

Based on the 24-week, prospective, non-interventional, observational study, A_1_chieve®, we investigated how health-related quality of life (HRQoL) changed, and the predictors of such changes, in Chinese people with type 2 diabetes mellitus (T2DM) after starting with, or switching to, biphasic insulin aspart 30 (BIAsp 30).

**Methods:**

In total, 8,578 people with T2DM starting treatment with, or switching to, BIAsp 30 were recruited from 130 urban hospitals in China. HRQoL was assessed at baseline and 24 weeks using the EuroQol-5 dimensions (EQ-5D) questionnaire. Descriptive statistics, paired *t*-test, and chi-square test were conducted and the linear ordinary least squares regression model was used to determine predictors for changes in EQ-5D score.

**Results:**

Haemoglobin A_1c_ (HbA_1c_) decreased from 9.5% to 7.0% after 24 weeks. The reported HRQoL measured by the EQ-5D visual analogue scale score increased by 6.2 (p < 0.001) from 75.8 to 82.0, and EQ-5D index score increased by 0.018 (p < 0.001) from 0.875 to 0.893 for the cohort over 24 weeks. The percentage of patients reporting no problems in the mobility, pain/discomfort, and anxiety/depression dimensions of EQ-5D increased significantly (p < 0.001) from 88.4% to 91.4%, 77.3% to 82.8%, and 74.2% to 77.1%, respectively. Patients with higher HbA1c levels at baseline, major hypoglycaemia or micro-complications exhibited significantly larger changes in EQ-5D scores than those with lower baseline HbA1c levels, without major hypoglycaemia or micro-complications after controlling for demographics and other baseline characteristics.

**Conclusions:**

BIAsp 30 treatment was associated with improved glycaemic control and HRQoL in T2DM patients in China. Patients with worse health conditions were more likely to experience larger improvements in HRQoL than those with better health conditions.

**Trial registration:**

ClinicalTrials.gov, NCT00869908.

**Electronic supplementary material:**

The online version of this article (doi:10.1186/s12955-014-0137-9) contains supplementary material, which is available to authorized users.

## Background

Globally, the number of people with diabetes has increased at an alarming level, and diabetes is placing a heavy economic burden on families and healthcare systems. The number of people with diabetes was estimated at more than 371 million in 2012, and is expected to be 551.9 million in 2030 [1]. China has become the country with the largest number of people with diabetes in the world. The most recent study estimated that the prevalence of diabetes among a representative sample of Chinese adults was 11.6% and the prevalence of pre-diabetes was 50.1%, which corresponded to 113.9 million and 493.4 million people, respectively, in 2010 [2]. The Chinese Diabetes Society of the Chinese Medical Association and International Diabetes Federation estimated that 13% of total medical expenditures in China were directly caused by diabetes in 2010 [3].

Diabetes is a debilitating disease characterized by deficiencies in insulin secretion, insulin action, or both, leading to chronic hyperglycaemia [4]. Insulin treatment is the inevitable choice for people with type 2 diabetes (T2DM) as diabetes progresses. It is typically used after glycaemic control fails or is not maintained with lifestyle changes and combinations of oral anti-diabetic medications [5]. Insulin treatment can improve glycaemic control, prevent the development of long-term complications of diabetes [6], and influence patients’ quality of life [7].

There are a few studies concerning the impacts of insulin use on patients’ health-related quality of life (HRQoL), with the impacts recorded ranging from positive [8-10] to negative [11-13]. There are no studies regarding whether insulin therapy improves or decreases patients’ quality of life in a Chinese setting. The purpose of this study was to assess how HRQoL changed, and the predictors of such changes, after starting with, or switching to, biphasic insulin aspart 30 (BIAsp 30, 30% soluble insulin aspart, 70% protamine-crystallized insulin aspart) over a 24-week period among people with T2DM in China using Chinese subgroup data from the A_1_chieve®, study [14].

## Methods

### Study design

A_1_chieve® was a 24-week, international, prospective, multicentre, non-interventional, observational study of people with T2DM in non-Western countries who had begun using basal insulin detemir, bolus insulin aspart and premixed insulin BIAsp 30, alone or in combination [14]. It was the largest observational study ever conducted in insulin therapy and was carried out in 28 countries across four continents (Asia, Africa, South America and Europe). Individuals with type 2 diabetes with no prior history of using the study insulins previously, and who had been started on one of the insulins in the 4 weeks prior to the study start are eligible for this study. People with hypersensitivity to the study insulins or excipients, and women who were pregnant, breast feeding, or who intended to become pregnant within 6 months of the study are excluded. The therapies were prescribed by the physicians in the course of normal clinical practice and treatment demand rather than randomly assigned by the researchers. The study was conducted in accordance with the Declaration of Helsinki. The ethics committee approval was obtained for each country, and all participants gave written, informed consent prior to their inclusion in the study. In China, central ethics committee approval was performed in China-Japan Friendship Hospital. The coordinating sites accept the central ethics committee approval or further conduct the ethics committee approval by ethics committee of their own hospitals (Additional file 1).

The Chinese cohort that either started (6,612) or were switched (1,966) to BIAsp 30 in the A_1_chieve® study consisted of 8,578 people with T2DM from 130 urban hospitals in China. They were recruited between January 2009 and June 2010, and had an average observation period of six months. Approval from ethics committees were obtained at all the study sites.

### Clinical endpoints

Clinical endpoints including safety and effectiveness outcomes were evaluated. Safety assessment included the incidence of serious adverse drug reactions (SADRs), including major hypoglycaemic events, the change in number of hypoglycaemic, the change in number of nocturnal hypoglycaemic event, and the number of adverse drug reactions (ADRs) from baseline to final visit. Effectiveness assessments included change in Haemoglobin A1c (HbA1c), fasting plasma glucose (FPG), postprandial plasma glucose (PPG), body weight between baseline and interim and final visits, and change in systolic blood pressure (SBP) and lipid profile at the final visit.

### HRQoL measurement

The HRQoL was measured by the Chinese version of EQ-5D questionnaire at baseline and after 24 weeks of therapy. The EQ-5D consists of a descriptive system of five dimensions: mobility, self-care, usual activities, pain/discomfort, and anxiety/depression. Each of the five dimensions can take one of three responses recording different level of severity: no problems, some or moderate problems and extreme problems. These responses could be converted into a single utility value using the EQ-5D preference weights elicited from general population samples. The EQ-5D also includes a visual analogue scale (VAS) recording the respondents’ direct valuation of their current HRQoL state on a graduated (0 − 100) scale with higher scores for higher HRQoL [15]. The Chinese version of the EQ-5D was obtained from the EuroQol Group [16]. Its validity and reliability have been assessed in mainland China [17-19] and it has been used for studies of different populations in mainland China [20-22].

### Statistical analyses

Descriptive analysis and multivariable regression were performed using SAS (Version 9.1.3, SAS Institute Inc., NC 27513-2414, USA). The change from baseline to 24 weeks in clinical endpoints, HRQoL with the EQ-5D VAS, and health utility value as continuous variables, were analysed with the Wilcoxon signed-rank test. The UK preference weights were used for calculation of EQ-5D utility value because Chinese preference weights were still to be established.

The change in the percentage of people reporting no problem in EQ-5D descriptive dimensions was analysed with a chi-square test. For descriptive analysis, the total cohort was divided into subgroups of insulin-naïve people (those not taking insulin therapy at baseline) and previously insulin-experienced people (current insulin users). Linear OLS regression was further employed to explore predictors of the changes in EQ-5D score. Independent variables included patients’ demographics (age and sex), health conditions (macro-complications, micro-complications, duration of diabetes, body mass index (BMI), HbA_1c_, SBP, total cholesterol, high-density lipoprotein (HDL) and low-density lipoprotein (LDL)), and other related indicators (previous insulin experience, total hypoglycaemia and major hypoglycaemia) at baseline.

## Results

### Demographics and characteristics of respondents

Among the 8,578 people with T2DM, 1,966 (22.9%) were in the insulin-experienced group and 6,612 (77.1%) in the insulin-naïve group. The average age was 54.9 (±14.4) years, and BMI was 24.7 (±3.3) kg/m^2^ for the total cohort. The average duration of diabetes was 6.0 (±6.0) years, with 9.3 (±6.8) for the insulin-experienced group and 5.0 (±5.4) for the insulin-naïve group (Table 1).

**Table 1:** **Characteristics of A**_**1**_ **chieve** **®** **study respondents in insulin-naïve and insulin-experienced groups, and in the total cohort**

		**Insulin experienced N = 1966**	**Insulin naïve N = 6612**	**Total cohort N = 8578**
Gender	Male, %	1104 (56.2)	3786 (57.3)	4890 (57.0)
Age	Years, mean (sd)	56.7 (15.2)	54.3 (14.2)	54.9 (14.4)
Weight	Kg, mean (sd)	68.2 (11.2)	68.1 (11.6)	68.1 (11.5)
BMI	Kg/m^2^, mean (sd)	24.8 (3.3)	24.6 (3.3)	24.7 (3.3)
Duration of diabetes	Years, mean (sd)	9.3 (6.8)	5.0 (5.4)	6.0 (6.0)

### Clinical endpoints

Blood glucose control measures improved markedly in both insulin-naïve and prior insulin users after 24 weeks of therapy with BIAsp 30. HbA_1c_ decreased from 9.5% to 7.0% for the total cohort, with a decrease from 9.1% to 7.0% for prior insulin users and a decrease from 9.6% to 7.0% for the insulin-naïve group. From a similar baseline measure, body weight of the two groups increased slightly by 0.3 kg during the therapy. No major hypoglycaemia was observed during the study, and reported hypoglycaemia rates (including overall, nocturnal and minor hypoglycaemia) decreased in the total cohort and in both subgroups. All of these results indicated BIAsp 30 could improve blood glucose control without increasing the risk of hypoglycaemia (Table 2). Indicators including FPG, PPG, SBP, LDL and HDL changed favourably, and no SADR was reported during the study period [23].

**Table 2:** **HbA**_**1c**_ **, body weight and hypoglycaemia in insulin-naïve and insulin-experienced patients, and in the total cohort**

		**Insulin-experienced, N = 1,966**	**Insulin-naïve, N = 6,612**	**Total cohort, N = 8,578**
HbA_1c_ (%)	N	1056	3386	4442
	Baseline	9.1 (2.3)	9.6 (2.2)	9.5 (2.3)
	Week 24	7.0 (1.1)	7.0 (1.0)	7.0 (1.0)
	Change	-2.0 (2.2)	-2.7 (2.2)	-2.5 (2.2)
	P value	<0.001	<0.001	<0.001
Body weight (kg)	N	1459	4600	6059
	Baseline	68.7 (11.2)	68.5 (11.3)	68.5 (11.3)
	Week 24	69.2 (10.9)	68.8 (10.8)	68.9 (10.8)
	Change	0.5 (3.0)	0.3 (3.1)	0.3 (3.1)
	P value	<0.001	<0.001	<0.001
Overall hypoglycaemia (events/patient year)	N	1966	6612	8578
	Baseline	6.12	1.2	2.32
	Week 24	2.14	1.37	1.54
Nocturnal hypoglycaemia (events/patient year)	N	1966	6612	8578
	Baseline	1.65	0.26	0.58
	Week 24	0.42	0.24	0.28
Major hypoglycaemia (events/patient year)	N	1966	6612	8578
	Baseline	0.37	0.09	0.15
	Week 24	0.00	0.00	0.00
Minor hypoglycaemia (events/patient year)	N	1966	6612	8578
	Baseline	5.75	1.10	2.17
	Week 24	2.14	1.37	1.54

Data are mean (sd), n or incidence.

### Quality of life

#### Quality of life in the total cohort

As measured by VAS from the EQ-5D (on a scale of 0–100), reported QoL of the total cohort increased by 6.2 from 75.8 at baseline to 82.0 at 24 weeks (p < 0.001). The health utility value (on a scale of 0–1) increased by 0.018 from 0.875 at baseline to 0.893 at 24 weeks (p < 0.001).

The increased percentages of people reporting no problem on the descriptive EQ-5D dimensions indicated that there were improvements of HRQoL after BIAsp 30 treatment. The percentages of patients reporting no problems in three of the five dimensions of EQ-5D—mobility, pain/discomfort and anxiety/depression—increased significantly from 88.4% to 91.4% (p < 0.0001), 77.3% to 82.8% (p < 0.0001) and 74.2% to 77.1% (p = 0.002) after 24 weeks, respectively. There was no statistical significance found in the percentage of patients who reported no problems in self-care or who reported no problems in usual activities (Table 3).

**Table 3:** **Quality of life in insulin-naïve and insulin-experienced patients, and in the total cohort**

		**Insulin-experienced, N = 1,966**	**Insulin-naïve, N = 6,612**	**Total cohort, N = 8,578**
EQ-5D VAS (Scale 0-100)	N	1390	4713	6103
	Baseline	75.3 (14.1)	75.9 (13.4)	75.8 (13.6)
	Week 24	82.0 (11.3)	82.0 (10.3)	82.0 (10.6)
	Change	6.7 (15.8)	6.1 (14.4)	6.2 (14.7)
	P value	<0.001	<0.001	<0.001
QoL UK (Scale 0-1)	N	1406	4814	6220
	Baseline	0.851 (0.190)	0.882 (0.176)	0.875 (0.179)
	Week 24	0.886 (0.170)	0.896 (0.157)	0.893 (0.160)
	Change	0.035 (0.228)	0.014 (0.218)	0.018 (0.221)
	P value	<0.001	<0.001	<0.001
Mobility dimension	N	1416	4843	6259
No problems with walking (%)	Baseline	1197 (84.5)	4333 (89.5)	5530 (88.4)
	Week 24	1289 (91.0)	4433 (91.5)	5722 (91.4)
	P value	<0.0001	0.0005	<0.0001
Self-care dimension	N	1415	4845	6260
No problems with self-care (%)	Baseline	1301 (91.9)	4475 (92.4)	5776 (92.3)
	Week 24	1280 (90.5)	4483 (92.5)	5763 (92.1)
	P value	0.1635	0.7584	0.6655
Usual activities dimension	N	1415	4841	6256
No problems with performing usual activities (%)	Baseline	1222 (86.4)	4316 (89.2)	5538 (88.5)
	Week 24	1236 (87.3)	4272 (88.2)	5508 (88.0)
	P value	0.4361	0.1578	0.4043
Pain/discomfort dimension	N	1411	4832	6243
No pain or discomfort (%)	Baseline	1007 (71.4)	3820 (79.1)	4827 (77.3)
	Week 24	1138 (80.7)	4031 (83.4)	5169 (82.8)
	P value	<0.0001	<0.0001	<0.0001
Anxiety/depression dimension	N	1413	4837	6250
Not anxious or depressed (%)	Baseline	1014 (71.8)	3624 (74.9)	4638 (74.2)
	Week 24	1067 (75.5)	3749 (77.5)	4816 (77.1)
	P value	0.0236	0.0028	0.0002

Data are mean (sd), n or percentage.

#### Quality of life for prior insulin-experienced and insulin-naïve subgroups

Quality of life improved in both insulin-experienced and insulin-naïve patients. Baseline EQ-5D VAS scores were similar for both prior insulin-experienced and insulin-naïve subgroups (75.3, 75.9). There was a significant increase in both subgroups after 24 weeks (+15.8, +14.4, p < 0.001). The baseline health utility value of the insulin-experienced group (0.851) was lower than that of the insulin-naïve group (0.882). After 24 weeks, the health utility value of the insulin-experienced and insulin-naïve groups increased by 0.035 (p < 0.001) and 0.014 (p < 0.001) resulting in a similar health utility value between the two groups.

The percentages of patients reporting no problems in dimensions of mobility, pain/discomfort and anxiety/depression increased significantly from 84.5% to 91.0% (p < 0.0001), 71.4% to 80.7%(p < 0.0001) and 71.8% to 75.5% (0.0236), respectively, for the prior insulin-experienced group, and from 89.5% to 91.5% (p = 0.0005), 79.1% to 83.4%(p < 0.0001) and 74.9% to 77.5% (p = 0.0028), respectively, for the insulin-naïve group. Decrease in percentages of patients reporting no problems were seen in the self-care dimension for the prior insulin-experienced group (from 91.9% to 90.5%) and in the usual activities dimension (from 89.2% to 88.2%) for the prior insulin-naïve group, but neither change was statistically significant. There were similar percentages of patients reporting no problems across all other dimensions between the two groups (Table 3).

#### Linear OLS regression for the change in EQ-5D score

Patients with higher HbA_1c_ levels at baseline, having major hypoglycaemia or micro-complications exhibited significantly larger changes in EQ-5D scores than those with lower baseline HbA_1c_ levels, without major hypoglycaemia or micro-complications after controlling for demographics and other baseline characteristics. HDL and LDL at baseline were negatively associated with change in EQ-5D scores. Other variables such as age, sex, duration of diabetes, and patients’ prior insulin experience were not significantly associated with change in HRQoL (Table 4).

**Table 4:** **Multivariate linear regression for change in EQ-5D VAS score**

**Variables**	**Coefficient**	**P-value**
Intercept	5.4377	0.1041
Macro-complications (yes = 1, no = 0)	-0.6064	0.3188
Micro-complications (yes = 1, no = 0)	1.4226	0.0067
Duration of diabetes	0.02993	0.5239
Age	0.02403	0.2699
Male	-0.839	0.0944
BMI at baseline	-0.1454	0.0554
Pre_study treatment (Insulin naïve = 1, insulin users = 0)	-0.5474	0.3547
HbA_1c_ at baseline	0.3459	0.0013
SBP at baseline	0.0208	0.1661
Total cholesterol at baseline	0.4528	0.0817
HDL at baseline	-2.3537	<.001
LDL at baseline	-0.7543	0.0138
Total hypoglycaemia at baseline (yes = 1, no = 0)	0.01457	0.9628
Major hypoglycaemia at baseline (yes = 1, no = 0)	6.957	<.001

## Discussion

This was the first study examining the impact of BIAsp 30 on HRQoL of people with T2DM in China. The result showed that people with T2DM starting with, or switching to, BIAsp 30 experienced significantly increased HRQoL over 24 weeks. The findings of this study were consistent with previous studies [24,25] in other countries based on A_1_chieve® that evaluated how patients’ HRQoL changed after BIAsp 30 treatment.

The efficacy and safety of BIAsp 30 compared with other insulins were shown in randomized controlled trials [26-30], and the effectiveness of BIAsp 30 in near-routine clinical practice was demonstrated by observational studies [31,32]. This study extended the results from clinical outcomes of BIAsp 30 and added additional evidence for decision making by assessment of humanistic outcomes. HRQoL was considered as a multidimensional concept reflecting patients’ subjective perceptions of their physical, mental and social functioning [33]. Measuring HRQoL provided a way to know patients’ subjective perceptions of clinical practice, and allowed a comprehensive evaluation of the health intervention. There was evidence that proper assessment of HRQoL during healthcare management can result in improvements to the patients’ health [34].

BIAsp30 treatment could be the most likely factor for improvements in HRQoL in this study. After treatment of BIAsp30, the patients’ glycaemic control improved and rates of hypoglycaemic events decreased, and it is recognized both of these could lead to improvement in HRQoL [35,36]. However, since the A_1_chieve® was non-randomised and lacked a standardised treatment protocol, it should be noted that factors other than BIAsp 30 therapy itself could contribute to the improvements as well. The circumstances in which BIAsp 30 was started were unknown, and patients’ self-management activities might be enhanced. Concomitant medication and dietary intake were not controlled either [14].

In addition to the impact of BIAsp 30 therapy on HRQoL, this paper also examined predictors for such impacts. The results of multivariable linear regression showed patients with a higher HbA_1c_ level, major hypoglycaemia or micro-complications at baseline experienced a larger amount of change in their EQ-5D scores. This finding indicates that patients with worse health conditions at baseline were more likely to experience larger improvements of HRQoL than those with better health conditions.

There were several limitations in this study. First, because evaluation of HRQoL was based on the observational A_1_chieve® study, which was non-randomised and lacked a standardised treatment protocol, confounding factors such as improvement of life style might affect patients’ HRQol. Second, the UK preference weights used for utility calculations of EQ-5D in this study might differ from those of comparable Chinese weights and result in an inaccurate evaluation of change in HRQoL in Chinese people with T2DM. Moreover, although EQ-5D has been widely used in treatment evaluation for diabetes, disease-specific questionnaires are often regarded as more sensitive than generic measures such as EQ-5D for capturing the impact of treatment [6]. All of these issues leave room for future research.

## Conclusion

This study suggested that BIAsp 30 treatment was associated with improved glycaemic control and HRQoL in people with T2DM in China. Patients with worse health conditions were more likely to experience larger improvements of HRQoL than those with better health conditions.

## Additional file

Additional file 1:
**130 hospitals and EC information for A**_**1**_ **chieve study in China.**

## Abbreviations

EQ-5D: EuroQol-5 dimensions
HRQoL: Health-related quality of life
T2DM: Type 2 diabetes mellitus
BIAsp 30: Biphasic insulin aspart 30
SADR: Serious adverse drug reaction
ADR: Adverse drug reaction
FPG: Fasting plasma glucose
PPG: Postprandial plasma glucose
SBP: Systolic blood pressure
VAS: Visual analogue scale
BMI: Body mass index
HDL: High-density lipoprotein
LDL: Low-density lipoprotein
HbA_1c_: Haemoglobin A_1c_
