# Supplementary material for: Improvements in quality of life associated with biphasic insulin aspart 30 in type 2 diabetes patients in China: results from the A1chieve® observational study
Source: Health Qual Life Outcomes. 2014 Nov 26;12:137. doi: 10.1186/s12955-014-0137-9 (PMC4253979; doi:10.1186/s12955-014-0137-9)
Supplement: Additional file 1: — 130 hospitals and EC information for A 1 chieve study in China. [file 12955_2014_137_MOESM1_ESM.doc]

|  |  |  |  |  |
| --- | --- | --- | --- | --- |

| **Site No.** | **Hospital Name** | **IEC/IRB Name** |
| --- | --- | --- |
| **Accept central Ethics**  **Committee** | *China-Japan Friendship Hospital* | *Ethics Committee of China-Japan Friendship Hospital（Central Ethics Committee）* |
|  | *Qian’an county hospital of Tangshan City* | *Qian’an county hospital of Tangshan City* |
|  | *Yan An University Affiliated Hospital* | *Ec of Yan An University Affiliated Hospital* |
|  | *The 2nd Affiliated Hospital of the Fourth Military Medical University(Tangdu Hospital)* | *Ec of the 2nd Affiliated Hospital of the Fourth* |
|  | *Zhao mine hospital, Kailuan, Tangshan City* | *No EC committee, follow Central IEC’s opinion and approval* |
|  | *Yutian county Hospital of Tangshan City* | *No EC committee, follow Central IEC’s opinion and approval* |
|  | *Affiliated hospital of North China Coal Medicine College* | *No EC committee, follow Central IEC’s opinion and approval* |
|  | *Tangshan municipal hospital of traditional Chinese medicine* | *No EC committee, follow Central IEC’s opinion and approval* |
|  | *Worker Hospital of Tangshan City* | *No EC committee, follow Central IEC’s opinion and approval* |
|  | *#1 Hospital of Shijiazhuang City* | *No EC committee, follow Central IEC’s opinion and approval* |
|  | *People’s Hospital of Hebei province* | *EC of People’s Hospital of Hebei province/* |
|  | *#4 Hospital of Hebei Medical College* | *No EC committee, follow Central IEC’s opinion and approval* |
|  | *#3 Hospital of Hebei Medical College* | *EC of #3 Hospital of Hebei Medical College/* |
|  | *#2 hospital of Hebei Medical College* | *EC of #2 hospital of Hebei Medical College/* |
|  | *Peking Union Medical College Hospital under the Chinese Academy of medical sciences* | *EC of Peking Union Medical College Hospital under the Chinese Academy of medical sciences/* |
|  | *Beijing Anzhen Hospital,affiliated hospital of Capital Medical College* | *EC of Beijing Anzhen Hospital,affiliated hospital of Capital Medical College/* |
|  | *Beijing Electricity Hospital* | *EC of Beijing Electricity Hospital/* |
|  | *Beijing Fuxing Hospital* | *EC of Beijing Fuxing Hospital/* |
|  | *People’s Hospital of Changshou District, Chongqing City* | *People’s Hospital of Changshou District, Chongqing City/* |
|  | *Diabetes Hospital of Mudanjiang City/*  *#5, Hailin Road, Mudanjiang City*  *Ni Xiushi/* | *EC of Diabetes Hospital of Mudanjiang City/* |
|  | *#1 People’s Hospital of*  *Shanghai(Senile Dept.)and (Endocrine dept.)* | *EC of #1 People’s Hospital of Shanghai(Senile Dept.)/* |
|  | *Shanghai Changzheng Hospital/* | *EC of Shanghai Changzheng Hospital/* |
|  | *Central Hospital of Xuhui district, Shanghai/* | *EC of Central Hospital of Xuhui district, Shanghai/* |
|  | *#411 Hospital of China Liberation Army/* | *EC of #411 Hospital of China Liberation Army/* |
|  | *Public Hospital of Pudong new district, Shanghai/i* | *EC of Public Hospital of Pudong new district, Shanghai/* |
|  | *Shuguang Hospital/* | *EC of Shuguang Hospital/* |
|  | *East China Hospital, affiliated hospital of Fudan University/* | *EC of East China Hospital, affiliated hospital of Fudan University/* |
|  | *Jinshan Hospital, affiliated hospital of Fudan University/* | *EC of Jinshan Hospital, affiliated hospital of Fudan University/* |
|  | *Jinshan Branch hospital of #6 People’s Hospital/* | *EC of Jinshan Branch hospital of #6 People’s Hospital/* |
|  | *Hongqi Hospital of Mudanjiang city/* | *EC of Hongqi Hospital of Mudanjiang city/* |
|  | *#1 Hospital of Mudanjiang city/* | *EC of #1 Hospital of Mudanjiang city/* |
|  | *Longnan Hospital of Daqing city/* | *EC of Longnan Hospital of Daqing city/* |
|  | *#2 Hospital of Jilin University/* | *EC of #2 Hospital of Jilin University/* |
|  | *China-Japan Friendship Hospital of Jilin University/* | *EC of China-Japan Friendship Hospital of Jilin University/* |
|  | *Central Hospital of Dalian city/* | *No EC committee, follow Central IEC’s opinion and approval* |
|  | *Red-cross Hospital of Shenyang city/* | *No EC committee, follow Central IEC’s opinion and approval* |
|  | *#4 People’s Hospital of Shenyang city/* | *No EC committee, follow Central IEC’s opinion and approval* |
|  | *General Hospital of Shenyang Military Command/* | *EC of General Hospital of Shenyang Military Command/* |
|  | *People’s Hospital ofLi shui County,Nanjing city/* | *EC of People’s Hospital of Lishui County,Nanjing city/* |
|  | *People’s Hospital of Jiangning District/* | *EC of People’s Hospital of Jiangning District/* |
|  | *Jiangsu Province Govermental Hospital/* | *EC of Jiangsu Province Govermental Hospital/* |
|  | *Jiangsu Provincial Hospital of Integrated Traditional Chinese with Western Medicine/* | *EC of Jiangsu Provincial Hospital of Integrated Traditional Chinese with Western Medicine/* |
|  | *Nanjing Jiangbei People’s Hospital/* | *No EC committee, follow Central IEC’s opinion and approval* |
|  | *#1 People’s Hospital of Yangzhou city/* | *EC of #1 People’s Hospital of Yangzhou city/* |
|  | *Subei People’s Hospital/* | *EC of Subei People’s Hospital /* |
|  | *#1 Hospital of Xuzhou city/* | *No EC committee, follow Central IEC’s opinion and approval* |
|  | *Affiliated Hospital of Xuzhou Medical College/* | *EC of Affiliated Hospital of Xuzhou Medical College/* |
|  | *Central Hospital of Xuzhou city/* | *EC of Central Hospital of Xuzhou city/* |
|  | *#1 People’s Hospital of Yunnan Province/* | *EC of #1 People’s Hospital of Yunnan Province/* |
|  | *#3 People’s Hospital of Yunnan Province/* | *No EC committee, follow Central IEC’s opinion and approval* |
|  | *Yan’an Hospital of Kunming city/* | *No EC committee, follow Central IEC’s opinion and approval* |
|  | *#1 People’s Hospital, Kunming city/* | *No EC committee, follow Central IEC’s opinion and approval* |
|  | *Xiangya Hospital of Central South University/* | *EC of Xiangya Hospital of Central South University/* |
|  | *The Third Hopspital of Xiangya Central South University /* | *EC of The Third Hopspital of Xiangya Central South University /* |
|  | *#1 Hospital of Changsha city/* | *No EC committee, follow Central IEC’s opinion and approval* |
|  | *People’s Hospital of Nanshan District, Shenzhen city/* | *No EC committee, follow Central IEC’s opinion and approval* |
|  | *Shenzhen Municipal Hospital of Traditional Chinese Medicine/* | *No EC committee, follow Central IEC’s opinion and approval* |
|  | *People’s Hospital of Luohu District,Shenzhen City/* | *EC of People’s Hospital of Luohu District,Shenzhen City/* |
|  | *Central Hospital of Longgang District, Shenzhen City/* | *In research office for record* |
|  | *People’s Hospital of Bao’an District, Shenzhen city/* | *EC of People’s Hospital of Bao’an District, Shenzhen city* |
|  | *People’s Hospital of Shekou District, Shenzhen city/* | *No EC committee, follow Central IEC’s opinion and approval* |
|  | *Central Hospital of Zhengzhou city/* | *No EC committee, follow Central IEC’s opinion and approval* |
|  | *#1 Affiliated Hospital of Zhenzhou University/* | *Archived at research department* |
|  | *People’s Hospital of Zhenzhou city/* | *No EC committee, follow Central IEC’s opinion and approval* |
|  | *People’s Hospital of Henan province/* | *EC of People’s Hospital of Henan province/* |
|  | *2nd Hospital of Shanxi Medical College/* | *EC of Clinical Pharmacology Study Site of*  *2nd Hospital of Shanxi Medical College* |
|  | *People’s Hospital of Shanxi Province/* | *No EC committee, follow Central IEC’s opinion and approval* |
|  | *Yuhuangding Hospital of Yantai city/* | *EC of Yuhuangding Hospital of Yantai city/* |
|  | *#107 Hospital of Yantai city /* | *EC of #107 Hospital of Yantai city/* |
|  | *The 1st Affiliated hospital of Xi’an Jiaotong University/* | *EC of The 1st Affiliated hospital of Xi’an Jiaotong University/* |
|  | *Central Hospital of Xi’an city/* | *No EC committee, follow Central IEC’s opinion and approval* |
|  | *The 3rd Affiliated Hospital of Nanchang University/* | *No EC committee, follow Central IEC’s opinion and approval* |
|  | *the 3rd Hospital of Nanchang city/* | *No EC committee, follow Central IEC’s opinion and approval* |
|  | *Wujin People’s Hospital of Changzhou city/* | *EC of Wujin People’s Hospital of Changzhou city /* |
|  | *Changzhou Municipal Hospital of Traditional Chinese Medicine/* | *EC of Changzhou Municipal Hospital of Traditional Chinese Medicine/* |
|  | *The 1st People’s Hospital of Nantong city/* | *EC of The 1st People’s Hospital of Nantong city/* |
|  | *People’s Hospital of Hai’an County/* | *EC of th People’s Hospital of Hai’an County/* |
|  | *#3 People’s Hospital of Wenzhou city/* | *No EC committee, follow Central IEC’s opinion and approval* |
|  | *#3 Peole Hospital of Hangzhou City/* | *No EC committee, follow Central IEC’s opinion and approval* |
|  | *#1 People’s Hospital of Kunshan city/* | *EC of #1 People’s Hospital of Kunshan city/* |
|  | *Zhangjiagang Municipal Hospital of Traditional Chinese Medicine/* | *EC of Zhangjiagang Municipal Hospital of Traditional Chinese Medicine/* |
|  | *the 1st People’s Hospital of Changzhou city/* | *EC of the 1st People’s Hospital of Changzhou city/* |
|  | *People’s Hospital of Jiangyin city/* | *EC of People’s Hospital of Jiangyin city /* |
|  | *People’s Hospital of Taizhou city/* | *EC of People’s Hospital of Taizhou city /* |
|  | *Wuxi Municipal Hospital of Integrated Traditional Chinese with Western Medicine/* | *EC of Wuxi Municipal Hospital of Integrated Traditional Chinese with Western Medicine /* |
|  | *People’s Hospital of Sichuan Province/* | *EC of People’s Hospital of Sichuan Province /* |
|  | *Affiliated Hospital of Chengdu College of Traditional Chinese Medicine/* | *No EC committee, follow Central IEC’s opinion and approval* |
|  | *the 3rd People’s Hospital of Chengdu city /* | *No EC committee, follow Central IEC’s opinion and approval* |
|  | *Central Hospital of Chengdu Railways Bureau/* | *EC of Central Hospital of Chengdu Railways Bureau/*  *Luo Guang Ping* |
|  | *the 5th People’s Hospital of Sichuan Province/* | *the 5th People’s Hospital of Sichuan Province/*  *No EC committee, follow Central IEC’s opinion and approval* |
|  | *The general hospital of Chengdu Military Command of Chinese Liberation Army/* | *EC of The general hospital of Chengdu Military* |
|  | *The 9th People’s Hospital of Chongqing city /* | *EC of The 9th People’s Hospital of Chongqing city /* |
|  | *the 1st Affiliated Hospital of the 3rd Military Medical College/* | *EC of the 1st Affiliated Hospital of the 3rd Military Medical College/* |
|  | *the 2nd Affiliated Hospital of the 3rd Military Medical College/* | *EC of the 2nd Affiliated Hospital of the 3rd Military Medical College/* |
|  | *the 2nd People’s Hospital of Chongqing city/* | *EC of the 2nd People’s Hospital of Chongqing city/* |
|  | *Fuling Central Hospital, Chongqing city/* | *EC of Fuling Central Hospital, Chongqing city/* |
|  | *Chongqing Three Gorges Central Hospital/* | *EC of Chongqing Three Gorges Central Hospital/* |
|  | *Tianjin huanghe hospital* | *No EC committee, follow Central IEC’s opinion and approval* |
|  | *Tianjin armed police hospital* | *No EC committee, follow Central IEC’s opinion and approval* |
|  | *Tianjin first central hospital* | *No EC committee, follow Central IEC’s opinion and approval* |
|  | *Mentabolic diseases hospital,Tianjin Medical University* | *No EC committee, follow Central IEC’s opinion and approval* |
|  | *The Armed Police General Hospital of Chongqing/* | *Archived at research department* |
|  | *Shengjing Hospital, affiliated hospital of China Medical College/(South Lake Branch)* | *EC of Shengjing Hospital, affiliated hospital of China Medical College/* |
|  | *Shengjing Hospital, affiliated hospital of China Medical College(HuaXiang Branch)* | *EC of Shengjing Hospital, affiliated hospital of China Medical College* |
|  | *People’s Hospital of Liaoning Province/* | *EC of People’s Hospital of Liaoning Province/* |
|  | *The 4th Hospital of Harbin Medical College/* | *EC of The 4th Hospital of Harbin Medical College/*  *#31 Yinhang Street, Harbin city/*  *Zhu Liying* |
|  | *The Affiliated Hospital of Luzhou Medical College/* | *EC of the Affiliated Hospital of Luzhou Medical College/* |
|  | *The Affiliated Hospital of Liaoning TCM College/* | *EC of The Affiliated Hospital of Liaoning TCM College/* |
|  | *The 1st Hospital of Shanxi Medical College/* | *EC of The 1st Hospital of Shanxi Medical College/* |
|  | *the 1st Affiliated Hospital of Heilongjiang TCM College/* | *EC of the 1st Affiliated Hospital of Heilongjiang TCM College* |
|  | *the 2nd Affiliated Hospital of Zhejiang TCM College/* | *EC of the 2nd Affiliated Hospital of Zhejiang TCM College/* |
|  | *the 1st affiliated hospital of Medical collage of Zhangjiang University/* | *EC of the 1st affiliated hospital of Medical collage of Zhangjiang University/* |
|  | *Second hospital of Tianjin Medical University* | *EC of Second hospital of Tianjin Medical University* |
|  | *Qinhuangdao second hospital*  *Chengguan three Street,Changli county,Qinhuangdao* | *EC of Qinhuangdao second hospital* |
|  | *Qinhuangdao beidaihe hospital* | *EC of Qinhuangdao beidaihe hospital* |
|  | *Qinhuangdao* orthopaedics *hospital*  *Tiexinli,HaigangDistrict,Qinhuangdao* | *EC of Qinhuangdao guke hospital* |
|  | *Qinhuangdao Traditional Chinese Medicine Hospital* | *EC of Qinhuangdao Traditional*  *Qinhuangdao Traditional Chinese Medicine Hospital* |
|  | *Aire Force General Hospital,PLA* | *Ec of Aire Force General Hospital,PLA* |
|  | *The Hospital of Inner Mongolia North Heavy Industry Ltd CO.* | *Ec of the Hospital of Inner Mongolia North Heavy Industry Ltd CO.* |
|  | *The 3rd Affiliated Hospital of Inner Mongolia Medical College, Baogang Hospital* | *Ec of the 3rd Affiliated Hospital of Inner Mongolia Medical College, Baogang Hospital* |
|  | *E Er duosi Central Hospital* | *Ec of E Er duosi Central Hospital* |
|  | *The 4th Hospital of Baotou* | *Ec of the 4th Hospital of Baotou* |
|  | *Inner Mongolia Self-government Hospital* | *Ec of Inner Mongolia Self-government Hospital* |
|  | *Baotou Electric Power Central Hospital( The 2nd Affiliated Hospital of Baotou Medical College)* | *Ec of Baotou Electric Power Central Hospital( The 2nd Affiliated Hospital of Baotou Medical College)* |
|  | *Baotou central Hospital* | *Ec of Baotou central Hospital* |
|  | *Shanxi Provincial People’s Hospital* | *Ec of Shaanxi Provincial People’s Hospital* |
|  | *Beijing Haidian Hospital* | *Ec of Beijing Haidian Hospital* |
|  | *Beijing Friendship Hospital* | *EC of Beijing Friendship Hospital* |
|  | *The 306th Hospital of PLA* | *Ec of the 306th Hospital of PLA* |
|  | *#1 Hospital of Jiling University/*  *istrict, Changchun City* | *EC of #1 Hospital of Jiling University* |
